# Reconstitution in Proteoliposomes of the Recombinant Human Riboflavin Transporter 2 (SLC52A2) Overexpressed in *E. coli*

**DOI:** 10.3390/ijms20184416

**Published:** 2019-09-08

**Authors:** Lara Console, Maria Tolomeo, Matilde Colella, Maria Barile, Cesare Indiveri

**Affiliations:** 1 Department DiBEST (Biologia, Ecologia, Scienze della Terra) Unit of Biochemistry and Molecular Biotechnology, University of Calabria, Via P. Bucci 4C, 87036 Arcavacata di Rende, Italy; 2 Department of Biosciences, Biotechnology and Biopharmaceutics, University of Bari, via Orabona 4, 70126 Bari, Italy

**Keywords:** riboflavin, transport, proteoliposomes, SLC, FMN

## Abstract

Background: the *SLC52A2* gene encodes for the riboflavin transporter 2 (RFVT2). This transporter is ubiquitously expressed. It mediates the transport of Riboflavin across cell membranes. Riboflavin plays a crucial role in cells since its biologically active forms, FMN and FAD, are essential for the metabolism of carbohydrates, amino acids, and lipids. Mutation of the Riboflavin transporters is a risk factor for anemia, cancer, cardiovascular disease, neurodegeneration. Inborn mutations of *SLC52A2* are associated with Brown-Vialetto-van Laere syndrome, a rare neurological disorder characterized by infancy onset. In spite of the important metabolic and physio/pathological role of this transporter few data are available on its function and regulation. Methods: the human recombinant RFVT2 has been overexpressed in *E. coli*, purified and reconstituted into proteoliposomes in order to characterize its activity following the [^3^H]Riboflavin transport. Results: the recombinant hRFVT2 showed a Km of 0.26 ± 0.07 µM and was inhibited by lumiflavin, FMN and Mg^2+^. The Riboflavin uptake was also regulated by Ca^2+^. The native protein extracted from fibroblast and reconstituted in proteoliposomes also showed inhibition by FMN and lumiflavin. Conclusions: proteoliposomes represent a suitable model to assay the RFVT2 function. It will be useful for screening the mutation of RFVT2.

## 1. Introduction

Riboflavin, known as vitamin B2, represents one of the most heat-stable components of the B complex vitamins. Its name derives from the bright yellow color (flavin) and from the ribose presence in its structure. By contrast with bacteria, fungi and plants, which synthesize riboflavin starting from GTP and ribulose 5Pi [1], higher organisms lost the ability to synthesize this vitamin. However, in humans, riboflavin is essential for cell growth and functions, thus, it must be absorbed from the diet. Riboflavin absorption takes place mainly in the small intestine. The greatest contribution to riboflavin intake in Western diets is due to milk and dairy products. Meat and fish are also good sources of riboflavin as well as dark-green vegetables. Generally, a balanced diet meets the riboflavin recommended daily allowance (RDA) corresponding to 1.4 mg/day for an adult man [2]. Another source of this vitamin is the gut microbiota that releases free absorbable riboflavin [3]. By contrast, a very small amount of riboflavin, which comes from the diet, exists in the free form as isoalloxazine ring bound to a ribitol side chain while most of the vitamin is in the forms of FMN and FAD [4]. These have to be hydrolyzed to free riboflavin by pyrophosphatases and phosphatases located on the brush border membrane of the intestinal epithelium prior to absorption [5]. Then, riboflavin is taken up by enterocytes by transporters which have been hypothesized [6] and then studied in intact cell systems [7]. Since, the biologically active forms of riboflavin are FMN and FAD that are crucial for the metabolism of carbohydrates, amino acids and lipids, riboflavin transporters and FAD forming/hydrolyzing enzymes have to cooperate to ensure the maintenance of cellular flavoproteome and for distributing riboflavin to the various tissues [8]. In the intestinal cells, Riboflavin undergoes ATP-dependent phosphorylation to form FMN, most of which is further converted into FAD by ATP-dependent adenylation to allow the functioning of several metabolic pathways. A fraction of intracellular flavin cofactors is converted, by still not well-characterized hydrolases, into free riboflavin that can be transported through the basolateral membrane of the enterocytes into the plasma. Circulating riboflavin is bound both to albumin and, more tightly, to a sub-fraction of immunoglobulins [9]. This riboflavin flux through mammalian cell membranes occurs via specific transporters belonging to the SLC52 family. The three members of the SLC52 family show poor sequence similarity with the riboflavin transporter of *Saccharomyces cerevisiae*, or with the bacterial riboflavin transporters, RibU, impX or RibM [7]. This low degree of similarity made it difficult to identify the mammalian transporters which remained unknown for many years. After the SLC classification acknowledgment, the members were named SLC52A1, also reported in the literature as RFT1 or RFVT1; SLC52A2 also known as RFT3 or RFVT2 and SLC52A3 previously called RFT2 or RFVT3 [7]. Alteration of the balance of flavoproteome, which the RFVTs contribute to preserving, is causative of severe disease. Indeed, under pathological stress, humans are susceptible to developing riboflavin deficiency. Such a deficiency in pregnancy induces fetus abnormalities and has been indicated as a risk factor for anemia, cancer, cardiovascular disease, and neurodegeneration [4]. Moreover, inherited diseases related to riboflavin deficiency have been identified. The most well described is the Brown–Vialetto–Van Laere syndrome, a rare neurological disorder characterized by infancy onset sensorineural deafness and ponto-bulbar palsy [10]. Among the transporters, more than forty mutations of SLC52A2 have been associated with Brown–Vialetto–Van Laere syndrome [11,12,13,14,15,16,17]. In spite of its important metabolic role and its relevance for human health, few data are available on this transporter. Actually, it is known that the *SLC52A2* gene is located at the 8q24.3 locus and encodes a 445-amino acid protein which is ubiquitous with the highest expression in brain and salivary gland [8]. RFVT2 shows 86% and 44% identity with RFVT1 and RFVT3, respectively. The hydropathy profile analysis predicts the presence of 11 putative membrane-spanning domains in its structure with a big hydrophilic loop located between the sixth and the seventh transmembrane segments. As RFVT1, it is located at the basolateral cell membrane while RFVT3 is mainly located at the apical membrane of the intestinal cells [7]. Knowledge of the transport function of all the SLC52 family members derives from data obtained in intact cells. As with the other members of the family, RFVT2 performs a sodium independent transport of riboflavin. The transport function seems to be favored at physiological pH, but does not show strong pH dependence. A Km of 0.33 µM was reported for riboflavin transport [18]. The RFVT2-mediated uptake of riboflavin was reported to be strongly inhibited by lumiflavin, a riboflavin derivative, while FMN and FAD were reported as poor inhibitors [7,8,18]. No data are available on the regulation of the transport activity of RFVT2. Some studies, done prior to the identification of riboflavin transporters, suggested that the cell uptake of riboflavin might be regulated by the Ca^2+^/calmodulin pathway. The implication of the PKA and PKG pathways in the regulation of cell absorption of riboflavin was also proposed [6].

Experimental approaches for studying the biochemical function of the transporter without the interference of other cellular components is mandatory for understanding the link between mutations and altered activity. In this work, the recombinant human RFVT2 has been produced in bacteria for functional assay in proteoliposomes.

## 2. Results

### 2.1. Expression and Purification of Riboflavin Transporter 2 (RFVT2)

**Figure 1:**
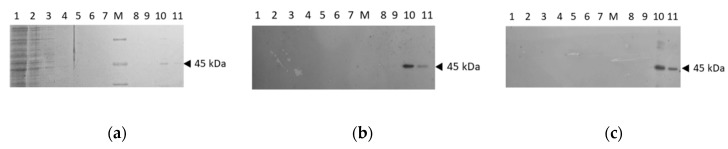
Purification and identification of the recombinant human RFVT2. (**a**) Solubilized bacterial proteins were loaded on a Ni^2+^-chelating chromatographic column. After column washing, RFVT2 was eluted using a buffer containing 0.1% C_12_E_8_, 200 mM NaCl, 10 mM Tris/HCl pH 8.0 and 50mM Imidazole. Fractions of 1 mL were collected and separated by sodium dodecyl sulfate polyacrylamide gel electrophoresis (SDS–PAGE) on 12% polyacrylamide gel and stained with Coomassie Blue. Lane 1 and 2 pass-through fractions, lane 3–7 washing fractions, lane 8–11 elution fractions; (**b**) Immunoblotting of the same samples of (a) using the anti-RFVT2 antibody whit a dilution of 1:2000; (**c**) Immunoblotting of the same samples of (a) using the anti-His antibody whit a dilution of 1:40000. Similar results were obtained in three different experiments.

In order to over-express the recombinant human protein, the cDNA coding for RFVT2 (SLC52A2) optimized for *E. coli* codon usage, was cloned into the pH6EX3 plasmid for transforming several *E. coli* strains, i.e., Rosetta (DE3), Lemo21(DE3) and Rosetta-gami 2(DE3). The most effective in protein expression was the Rosetta (DE3) strain. An anti-His antibody was exploited to test the expression of the 6-His-RFVT2 in bacterial lysates. The Isopropil-β-D-1-tiogalattopiranoside (IPTG) concentration and the time of growth were optimized for obtaining the best induction. 0.4 mM IPTG and 4 h after induction were used. Figure 1a shows a typical purification pattern of 6His-RFVT2 obtained after loading the solubilized bacterial proteins on a Ni^2+^-chelating resin and eluting the 6His-RFVT2 by imidazole. After applying the solubilized proteins to the column, the flow was stopped for 10 min to increase the binding of RFVT2 to the resin. Then the elution was performed with the washing buffer containing 0.1% C_12_E_8_, 200 mM NaCl, 10 mM Tris/HCl pH 8.0. The eluted fractions (pass-through) contained most of the bacterial proteins (Figure 1a fractions 1–2). Proteins disappeared in the next fractions eluted with the washing buffer (Figure 1a fractions 3–7). Then, 50 mM imidazole was added to the buffer. The purified protein was mainly recovered in fraction 10. The identity of the eluted protein was confirmed by anti-SLC52A2 antibody (Figure 1b) or anti-His antibody (Figure 1c). Interestingly, no staining was observed in the pass-through fraction indicating that, virtually, all the RFVT2 protein was bound to the resin. Both antibodies give strong staining of the protein in fraction 10. The staining decreased a lot in fraction 11. This behavior correlated well with the protein elution profile observed in Figure 1a. The apparent molecular mass calculated on the basis of standard proteins (Figure 1a line M) was 45 kDa. This mass well correlated to the theoretical mass of 46 kDa calculated for RFVT2.

### 2.2. Characterization of the Transport Activity of RFVT2

**Figure 2:**
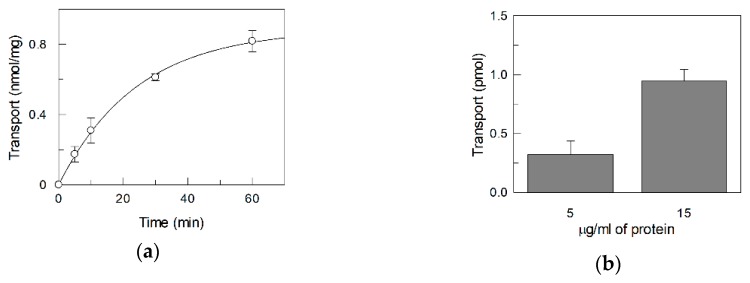
[^3^H]Riboflavin uptake into proteoliposomes reconstituted whit recombinant hRFVT2. (**a**) The transport measurement was started adding 0.1 µM [^3^H]riboflavin and stopped at the indicated times, as described in Materials and Methods. The values were corrected by subtracting the [^3^H]riboflavin taken up by diffusion as described in Materials and Methods. (**b**) Riboflavin uptake in proteoliposomes measured in 60 min at two different protein concentration. The values are means ± S.D. from three experiments.

For the functional assay, the purified 6His-RFVT2 was reconstituted into proteoliposomes by a methodology previously applied to SLC22A4 (OCTN1) and then adapted to other SLC transporters [19]. The reconstitution conditions were optimized for RFVT2. 0.6 (mg/mg) of detergent/phospholipid ratio and 15 µg/mL protein concentration gave the best transport activity measured as [^3^H]riboflavin uptake into proteoliposomes [20]. Protein-mediated transport was obtained by subtracting the diffusion as described in Materials and Methods. The data of the net [^3^H]riboflavin uptake into proteoliposomes are shown in Figure 2a. The time course shows a typical protein-mediated process than could be fitted into a first-order rate equation. Radioactivity equilibration was observed after 60 min while the [^3^H]riboflavin uptake increase was linear with the time up to at least 10 min. As further proof of the protein-mediated phenomenon, the transport was measured by reconstituting two different amount of protein. As shown in Figure 2b, the transport triplicates with a triplicate amount of reconstituted protein.

**Figure 3:**
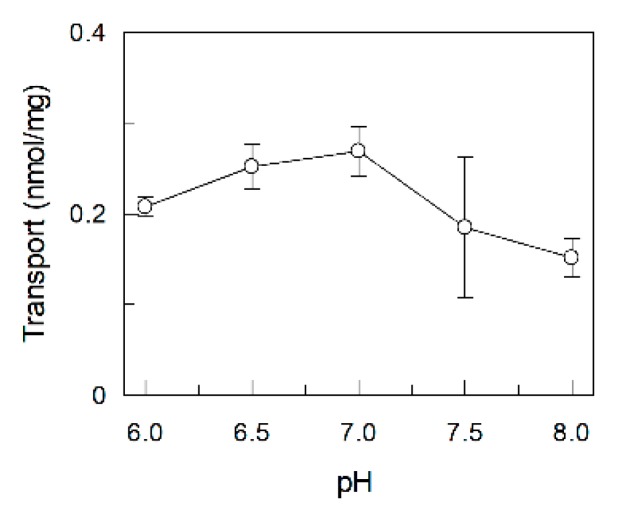
Dependence of the transport activity of hRFVT2 on the pH. Purified RFVT2 was reconstituted in proteoliposomes. [^3^H]Riboflavin uptake into proteoliposomes was performed at the indicated pH and stopped after 20 min as described in the Material and Methods section. The values are means ± standard deviation (SD) from three experiments.

Since the optimal activity of RFVT2 was reported at pH 7 in cell experiments, the dependence of the transport activity on the pH was tested in proteoliposomes. As shown in Figure 3, RFVT2 mediated transport was maximal at pH 6.5–7.0. It drastically decreased at more alkaline pH.

**Figure 4:**
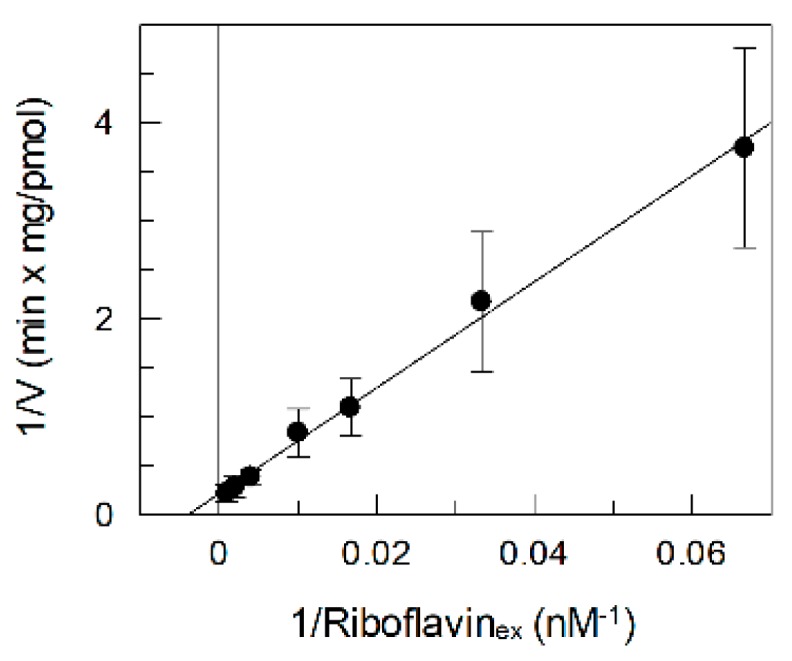
Dependence of the riboflavin transport on substrate concentration. [^3^H]Riboflavin was added to proteoliposomes at the indicated concentration. The transport measurement was stopped after 15 min. Data were plotted according to the Lineweaver–Burk equation. The values are means ± SD from three experiments.

For a better characterization of the recombinant transporter, the kinetics of the RFVT2 mediated transport has been studied in proteoliposomes by changing the riboflavin concentrations in the range reported in Figure 4. A Km of 0.26 ± 0.07 µM has been obtained which is similar to that found in intact cells (Figure 4) [18].

**Figure 5:**
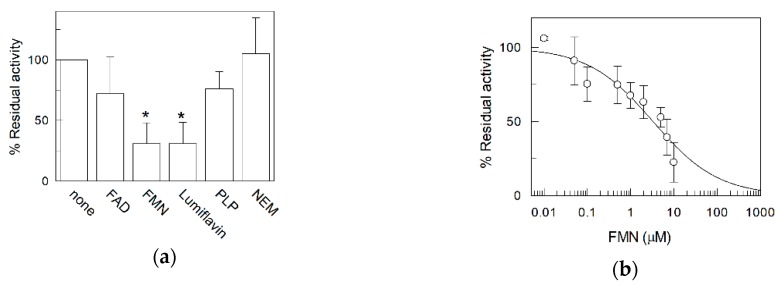
Effect of various compounds on riboflavin uptake. (**a**) Transport was measured as described in Materials and Methods. 10 µM FAD or 10 µM FMN or 100 µM lumiflavin or 10 mM Pyridoxal 5′-phosphate (PLP) or 1 mM N-Ethylmaleimide (NEM) were added 1 min before the labeled substrate. Percent of residual activity was calculated for each experiment with respect to the control sample (without added inhibitor, referred as 100%); (**b**) dose-response curve for the inhibition of RFVT2 transport activity by FMN was obtained adding FMN at the indicated concentrations 1 min before the labeled substrate. riboflavin transport was stopped after 30 min. The values are means ± SD from three experiments. Statistics was performed using Student’s *t*-test (* *p* < 0.05).

Some potential inhibitors tested on RFVT2 in intact cells were verified on the [^3^H]riboflavin uptake mediated by RFVT2. Figure 5a shows the effect of FAD, FMN, and lumiflavin, which are riboflavin derivatives on the [^3^H]riboflavin uptake. FAD did not show any effect, while 10µM FMN strongly inhibited the transport mediated by RFVT2. 100 µM lumiflavin was able to impair the activity of RFVT2. The effect of FMN was further characterized by a dose-response analysis. As shown in Figure 5b the IC_50_ for this compound was 3.3 ± 0.8 µM. Some other compounds which are able to bind specific amino acid residues of proteins have been tested. Pyridoxal 5′-phosphate (PLP) or N-Ethylmaleimide (NEM), which interact with amino groups of Lysine or thiol groups of cysteine, respectively, did not exert significant effects.

**Figure 6:**
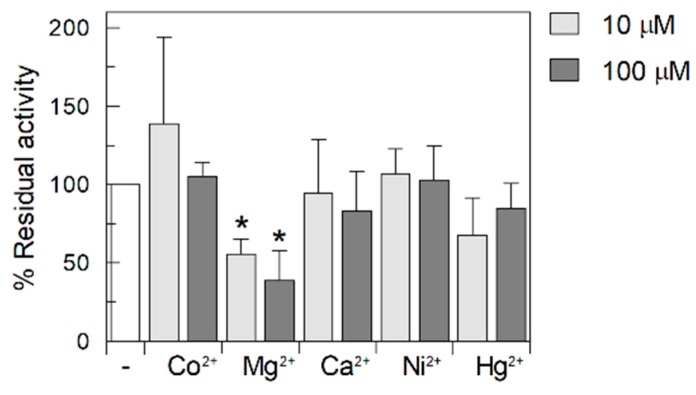
Effect of various divalent ion on riboflavin uptake. Transport was measured as described in Materials and methods. The two concentration of divalent ions were added 1 min before the labeled substrate. Riboflavin transport was stopped after 30 min. Percent of residual activity was calculated for each experiment with respect to the control sample (white bar) without added inhibitor and referred as 100%. The values are means ± SD from three experiments. Statistics was performed using Student’s *t*-test (* *p* < 0.05).

Since it was previously reported that the riboflavin transport might be influenced by Ca^2+^ [6], the effect of this and other divalent cations, i.e., Co^2+^, Ni^2+^, Hg^2+^ and Mg^2+^ on transport activity was characterized (Figure 6). Two concentrations were tested for each divalent ion. No statistically relevant data were obtained for Ca^2+^, Co^2+^, Ni^2+^ and Hg^2+^ while Mg^2+^ showed a potent inhibition at both the tested concentrations (Figure 6).

**Figure 7:**
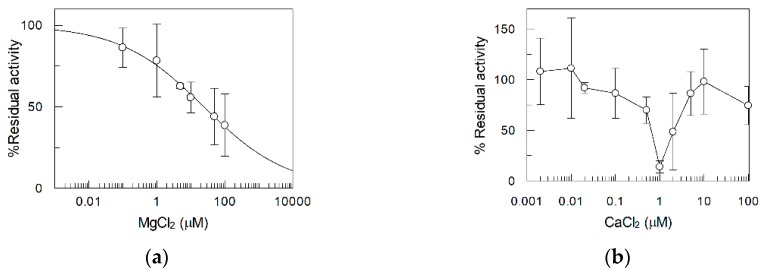
Dose-response curve for the inhibition of RFVT2 transport activity by MgCl_2_ (**a**) and CaCl_2_ (**b**)_._ It was obtained adding the divalent ions at the indicated concentrations 1 min before the labeled substrate. Riboflavin transport was stopped after 30 min. The values are means ± SD from three experiments.

The inhibition by Mg^2+^ was further investigated by a dose-response analysis (Figure 7a) from which the IC_50_ was derived: it was 24 ± 2.5 µM. A larger range of concentrations of the other cations was then tested to exclude possible effects. No effect could be detected except that in the case of Ca^2+^. As shown in Figure 7b, this cation exerted a quite complex behavior which was different from that of Mg^2+^. Ca^2+^ exerted a dual-mode effect which was detected as inhibition up to a concentration of 1 µM. The inhibition was reversed at higher concentrations up to a complete recovery of the transport activity at 10 µM.

**Figure 8:**
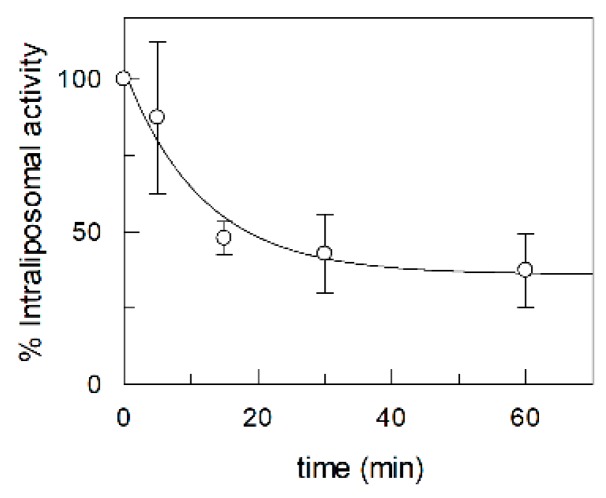
Efflux of riboflavin from proteoliposomes reconstituted with recombinant RFVT2. Proteoliposomes were incubated with [^3^H]riboflavin for 60 min. After this incubation, the external radiolabeled substrate was removed. The export of riboflavin from proteoliposomes was stopped at the indicated times and the residual intraliposomal radioactivity was measured as described in the Material and Method section. The values are means ± SD from three experiments.

Since under physiological conditions the transporter may also mediate the export of riboflavin [8], an efflux experiment was performed. As shown in Figure 8, the [^3^H]riboflavin effluxes from proteoliposomes with a behavior that was quite similar to that of the uptake. The efflux equilibrium was reached after about 60 min. This indicates that the same protein can catalyze both the processes.

### 2.3. Transport Assay of the Fibroblast Riboflavin Transporter

**Figure 9:**
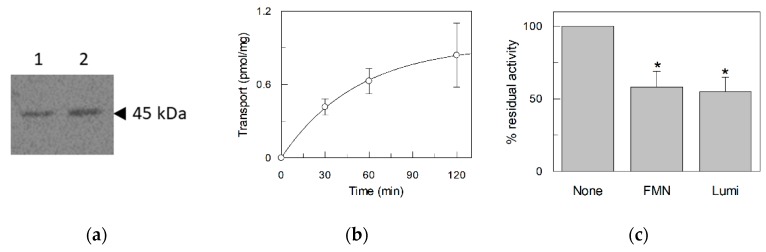
Native RFVT2 from primary human dermal fibroblasts. (**a**) Western Blot analysis of Native RFVT2. Protein extract from primary human dermal fibroblasts was separated by SDS–PAGE on 12% polyacrylamide gel and subjected to WB analysis using the anti-RFVT2 antibody whit a dilution of 1:2000. 50 µg and 60 µg of total protein extract from fibroblast were loaded in line 1 and 2 respectively; (**b**) time course of native RFVT2 extracted from primary human dermal fibroblasts and reconstituted in proteoliposomes. The transport measurement was started adding 0.1 µM [^3^H]riboflavin and stopped at the indicated times, as described in Materials and Methods; (**c**) Effect of FMN and lumiflavin on riboflavin uptake mediated by native RFVT2. The riboflavin transport was stopped after 120 min. The values are means ± SD from three experiments. Statistics was performed using Student′s *t*-test (* *p* < 0.05).

As a comparison of the data obtained with the recombinant human protein with a native protein, the riboflavin transporter was extracted from primary human dermal fibroblasts from healthy individuals. These cells indeed contain the RFVT2 as demonstrated by the Western Blot in Figure 9a. After extraction of the membrane proteins with non-ionic detergent [21,22] and reconstitution into proteoliposomes, the [^3^H]riboflavin uptake was measured (Figure 9b). The uptake was slower than that measured with the recombinant protein and reached the equilibrium at 120 min. The native protein was inhibited by FMN and lumiflavin as the recombinant protein confirming that the transport process is mostly mediated by the RFVT2 (Figure 9c).

### 2.4. Homology Structural Model of RFVT2

**Figure 10:**
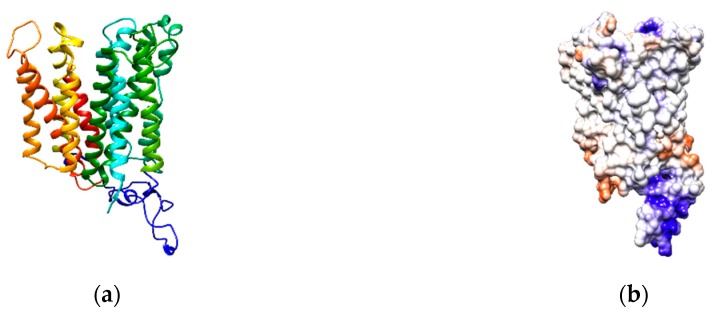
Structural model of RFVT2. (**a**) Lateral view of the ribbon diagram of the RFVT2. The protein is constituted by 11 transmembrane α-helices and a big intracellular loop which connect the transmembrane segment 6 with the transmembrane segment 7. The homology model was constructed by the SWISS-MODEL tool using the crystallographic structure of human Equilibrative Nucleoside Transporter 1 (ENT1) (PDB code 6OB6) as a template. (**b**) Columbic surface coloring of a space filled diagram of RFVT2 homology model. Molecular graphics was performed with UCSF Chimera [24].

The homology model of RFVT2 was built by Swiss model server [23] using the 3D structure of SLC29A1 (PDB code 6OB6) as a template. The protein consists of 11 α-helices crossing the membrane and 2 large hydrophilic loops (Figure 10). Interestingly one of the 2 loops that is highlighted in blue in Figure 10a, contains several negatively charged amino acids. These amino acids cluster in the middle of the loop as evidenced in Figure 10b that shows the coulomb charge distribution of the RFVT2 surface.

## 3. Discussion

The study of the riboflavin transporter RFVT2 is hampered by the lack of a suitable experimental model in which the human transporter could be studied in terms of transport function and structure/function relationships. Indeed, intact cell systems are used for measuring transport but they present several inconveniences mainly caused by the interferences due to the presence of other systems which can transport the same molecules and/or to the presence of intracellular enzyme pathways that degrade the transported molecules converting them into different chemical species. These problems are particularly challenging in the case of riboflavin transport since at least three riboflavin transporters are known that can be present into the same cell membrane. Then, riboflavin can be converted into other compounds by well-known intracellular enzyme pathways [8]. Thus, an experimental model allowing the detection of the transport process in the absence of interferences would be extremely useful. A model of choice is the proteoliposome system carrying the transporter obtained by recombinant methodology. Such a procedure was previously used for several transporters of the plasma membrane or of intracellular organelle membranes [25,26,27,28]. This approach reduces or eliminates the interferences and guarantees measuring a single transporter activity. No data obtained with such a model were available so far on riboflavin transporters either from plasma or from other membranes. Therefore, we have pointed out a procedure of over-production of the RFVT2 transporter by expression in bacteria. The purified transporter was then reconstituted into proteoliposomes for functional studies. Several parameters were measured that indicate that the transport activity detected indeed corresponds to the previously described RFVT2 transport system in intact cell systems. Parameters such as the Km or the pH dependence of the reconstituted transporter overlapped those of the transporter described in cells [7]. Additional properties of the riboflavin transporter have been revealed by the proteoliposome system. Very interestingly, the inhibition by FMN was clearly assessed. The IC_50_ revealed that inhibition is much stronger than that described in intact cells. This difference could be due to the presence in cells of other transporters which are not inhibited by FMN thus reducing the observable effect on RFVT2. Novel and interesting results were obtained on inhibition by divalent cations. We started this investigation from previous data describing the interrelations of the riboflavin transporter with calcium metabolism (pathways) [6]. It was found that Ca^2+^ interacts with the transport causing inhibition at a low concentration up to 1 µM and then recovery of activity at higher concentrations. These effects, which cannot be clearly ascribed to specific amino acid residues at this stage, can be anyway correlated to the grouping of acidic amino acid residues in a protein domain constituted by the large loop observed in the structure (Figure 10). In this area, a surface negative charge is also evident which may be involved in calcium (or Mg^2+^ binding). Indeed, the inhibition by Mg^2+^ was also a novel finding described for RFVT2 in proteoliposomes. The Km for riboflavin of the recombinant transporter, measured in this work, was very similar to that reported for the same transporter in the cell system. The comparison of the data of these Km values, i.e., 0.26 and 0.33 µM for the recombinant and the native transporters, respectively, let us to hypothesize that the sidedness of the transporter in proteoliposomes corresponds to that of the cell membrane. This finding is common to other transporters reconstituted with the same procedure. The slow detergent removal used in this method, facilitates a single direction of the protein that, very likely, corresponds to that of the native membrane. This is due to the asymmetric structure of transporters that are forced to assume a single orientation in the micelles formed at the beginning of the reconstitution procedure (as described in the Materials and Methods section). This is due to the relatively small radius of the micelles [29]. However, the actual orientation will be confirmed only when the complete kinetic characterization and the structure/function relationships of the transporter will be performed in further studies. Finally, RFVT2 can also catalyze the efflux of riboflavin. This is in agreement with the function of this or other members of the family in polarized epithelia from which riboflavin must be exported into the plasma for distribution to other tissues. Of great interest is the application of the experimental model set up to the transport assay of the fibroblast transporter. Indeed, fibroblasts are the cell systems carrying transporters from patients, that could be tested by a relatively simple and reproducible method. This also indicates that RFVT2 from other cell types could be reconstituted by a similar procedure. Interestingly, the proteoliposome system will then be used for characterizing RFVT2 natural variants which will be reproduced by site-directed mutagenesis.

## 4. Materials and Methods

### 4.1. Overexpression

The *E. coli* optimized cDNA coding for human RFVT2 (GenBank NM NM_024531) was purchased by GenScript and subcloned in the pH6EX3 expression vector. The sequence of the resulting recombinant plasmid, encoding a fusion protein corresponding to the hRFVT2 carrying an N-terminal 6-Histidine tag, was verified by sequencing. Chemically competent *E. coli* Rosetta(DE3) was transformed using the hRFVT2–pH6EX3 construct. After plate selection, colonies, carrying the recombinant plasmid, were inoculated in 100 mL of LB medium and cultured overnight at 37 °C. The pre-culture was diluted (1:10) in 1 L of fresh LB medium and incubated at 37 °C until reaching the OD of 0.6. The expression of hRFVT2 was induced adding 0.4 mM IPTG. After 4 h cells were harvested and centrifuged. The bacterial pellet was suspended in a buffer contemning NaCl 200 mM and 50 mM Hepes/Tris pH 7.5. Cells were disrupted by mild sonication at 4 °C.

### 4.2. Purification

The bacterial lysate was centrifuged at 12,000× *g* for 10 min at 4 °C; The resulting pellet containing the inclusion bodies was solubilized with a buffer made of 3 M urea, 0.8% Sarkosyl, 200 mM NaCl, 10 mM Tris/HCl pH 8.0 and centrifuged at 12,000× *g* for 10 min at 4 °C. The supernatant was applied onto a His-select column pre-conditioned with 10 mL of a buffer containing 0.1% Sarkosyl, 200 mM NaCl, 10 mM Tris/HCl pH 8.0. The column was washed with 7 mL of a buffer containing 0.1% C12E8, 200 mM NaCl, 10 mM Tris/HCl pH 8.0. The elution was performed adding 3 mL of the same buffer supplemented with 50 mM imidazole [30].

### 4.3. Reconstitution of RFVT2 into Liposomes

In the reconstitution procedure, mixed micelles containing detergents, recombinant proteins, and phospholipids are formed. This mixture is then incubated under rotatory stirring with a hydrophobic resin, in order to slowly remove the detergents and to allow the formation of proteoliposomes. The standard composition for reconstitution was: 400 μL of purified hRFVT2, 60 μL of 10% C12E8, 100 μL of 10% egg yolk phospholipids in the form of sonicated liposomes, 70 μL of 200mM Tris/HCl at 7.0 in a final volume of 700 μL. Reconstitution was achieved by removing the detergent from the previously described mix by incubation with 0.55 g Amberlite XAD-4 under rotatory stirring at room temperature for 90 min [31,32,33].

### 4.4. Transport Measurements

Proteoliposomes (550 μL) were passed through a Sephadex G-75 column (0.7 cm diameter × 15 cm height) pre-equilibrated with a buffer containing 20 mM Tris/HCl at pH 7.0 and 30 mM NaCl. The eluate was divided in aliquots of 100 μL. Transport was started by adding the [^3^H]riboflavin to the proteoliposome and stopped by passing the samples through pirces columns (0.6 cm diameter × 8 cm height) containing Sephadex G-75 to remove the radioactive substrate which is not entered into proteoliposome. The same procedure was performed also for the control sample containing liposome without hRFVT2. The experimental values were corrected by subtracting sole liposome radioactivity normalized respect to the liposome internal volume. The internal volume was measured as previously described by [34]. For efflux measurements, proteoliposomes were preloaded with [^3^H]riboflavin by incubation for 60 min. After incubation, the non-incorporated radioactive substrate was removed by passing the proteoliposome through a column containing Sephadex G-75. The time course of efflux was stopped at the indicated time intervals by passing the samples through pirces columns. The efflux from proteoliposomes was corrected as in the case of uptake, for the diffusion from liposomes without incorporated proteins. For native protein reconstitution, a pellet of primary human dermal previously-stored at −80 °C was solubilized whit a buffer containing 20 mM Tris/HCl at pH 7.0 and 200 mM NaCl and incubated in ice for 30 min. Than the solubilized fibroblasts were centrifuged at 12,000× *g* for 10 min and the supernatant (50 μg of total protein) was used to perform the proteoliposome reconstitution as described for the recombinant RFVTs.

### 4.5. Other Methods

Sodium dodecyl sulfate polyacrylamide gel electrophoresis (SDS-PAGE) was performed in the presence of 0.1 SDS according to Laemmli. The minigel system, sizes 8 cm × 10 cm × 0.75 mm, was used. Stacking gel and separation gel 5% and 12% acrylamide respectively (acrylamide/bisacrylamide ratio 30:0.2). Proteins were transferred to nitrocellulose membrane. Residual binding sites on the membrane were blocked by incubation with 0.5% bovine serum albumin (BSA) in buffer composed of NaCl 150 mM; 50 mM Tris-HCl, Tween20 0.05% pH 7, for 10 min and then incubated with a rabbit polyclonal anti-His (1:400000) or anti-SLC52A2 (dilution 1:2.000) in 0.5% BSA solution overnight at 10 °C [35].

## Abbreviations

FMN	Riboflavin 5′-monophosphate
FAD	Flavin adenine dinucleotide
RFVT2	Riboflavin Transporter 2 corresponding to SLC52A2
C12E8	Octaethylene glycol monododecyl ether
NEM	N-Ethylmaleimide
PLP	Pyridoxal 5′-phosphate
